# Re-demonstration without remediation – a missed opportunity? A national survey of internal medicine clerkship directors

**DOI:** 10.3402/meo.v19.25991

**Published:** 2014-12-10

**Authors:** Mary R. Hawthorne, Katherine C. Chretien, Dario Torre, Shobhina G. Chheda

**Affiliations:** 1 Department of Medicine, University of Massachusetts Medical School, Worcester, MA, USA; 2 Department of Medicine, George Washington University, Washington, DC, USA; 3 Department of Medicine, Drexel University College of Medicine, Philadelphia, PA, USA; 4 Department of Medicine, University of Wisconsin School of Medicine and Public Health, Madison, WI, USA

**Keywords:** remediation, failure, clerkship, clerkship failure, grade, clinical competence, medical knowledge, evaluation

## Abstract

**Background:**

Many different components factor into the final grade assigned for the internal medicine clerkship. Failure of one or more of these requires consideration of remedial measures.

**Purpose:**

To determine which assessment components are used to assign students a passing grade for the clerkship and what remediation measures are required when students do not pass a component.

**Methods:**

A national cross-sectional survey of Clerkship Directors in Internal Medicine (CDIM) institutional members was conducted in April 2011. The survey included sections on remediation, grading practices, and demographics. The authors analyzed responses using descriptive and comparative statistics.

**Results:**

Response rate was 73% (86/113). Medicine clerkships required students to pass the following components: clinical evaluations 83 (97%), NBME subject exam 76 (88%), written assignments 40 (46%), OSCE 35 (41%), in-house written exam 23 (27%), and mini-CEX 19 (22%). When students failed a component of the clerkship for the first time, 55 schools (64%) simply allowed students to make up the component, while only 16 (18%) allowed a simple make-up for a second failure. Additional ward time was required by 24 schools (28%) for a first-time failure of one component of the clerkship and by 49 (57%) for a second failure. The presence or absence of true remedial measures in a school was not associated with clerkship director academic rank, grading scheme, or percent of students who failed the clerkship in the previous year.

**Conclusions:**

Most schools required passing clinical evaluations and NBME subject exam components to pass the medicine clerkship, but there was variability in other requirements. Most schools allowed students to simply re-take the component for a first-time failure. This study raises the question of whether true remediation is being undertaken before students are asked to re-demonstrate competence in a failed area of the clerkship to be ready for the subinternship level.

Medical schools are charged with the responsibility of making sure that graduates are clinically competent to enter residency (1). To accomplish this goal, medical schools must first be able to identify learners who are struggling, implement programs that will address gaps, and then allow students to re-demonstrate to establish competence (2–5). Yates et al. identified that 10–15% of medical students struggle with performance in medical schools (6). Identification of struggling students has been the topic of many studies in the medical literature (7–11). Students may struggle because of poor knowledge base, inadequate communication skills, shyness, poor test-taking ability, substance abuse, depression, or medical illness (among other reasons) (12). When students underperform on one or more areas of the clerkship, clerkship directors are faced with the task of crafting remediation plans for those students (11). Some schools have policies that specify the remedial measures required for particular failures, while others may tailor remediation plans based on individual student needs (12–14). Durning et al. and Artino et al. described the use of a self-regulated learning perspective to help struggling students to identify their areas of weakness as well as the root causes of their struggles so that more effective remediation plans can be designed (11, 13, 15). Cleland et al., in a systematic review of the literature in 2013, found that most remediation interventions are aimed at improving performance on standardized tests and that there is no agreement on which interventions actually work (4).

Hauer et al. (3) identified a four-step process for remediation that includes 1) use of multiple assessment tools to identify deficiencies, 2) diagnosis of problems and development of an individualized learning plan, 3) provision of instruction that includes deliberate practice, feedback, and reflection, and 4) reassessment and certification of competence.

Medicine clerkship students at medical schools across the country are evaluated by a number of different methods, including clinical performance evaluations, OSCE's, written examinations, mini-CEX exams, written assignments, and assessments of professionalism. Failure of a component of the clerkship is one way in which a struggling learner may be identified.

Clerkship directors are then faced with the task of deciding how these students should remediate and/or re-demonstrate competence in the failed component (2–5). Little is known about what remediation strategies are used most in clinical clerkships. In this study, we sought to determine which strategies are used by clerkship directors at US and Canadian medical schools regarding remediation and re-demonstration of failed components of the medicine clerkship.

## Methods

In April 2011, Clerkship Directors in Internal Medicine (CDIM) conducted its annual, confidential, on-line survey of its institutional members. An invitation to submit questions for the 2011 survey was extended to all CDIM members in October 2010.

Questions were selected for inclusion based on topic priority, relevance to the mission of CDIM and question quality. Survey items were reviewed and edited for clarity by the CDIM Research Committee. Questions were then presented and approved by CDIM Council. The CDIM Research Committee pilot-tested the questions and additional revisions were made. The survey was sent out by email in April 2011 and up to three additional contacts were made to non-responders by email.

The survey consisted of five sections. One section contained demographic information of the respondents, including academic rank, age, gender, and academic role. One section asked about grading practices in the clerkship, including grading schema used on the clerkship (e.g., Honors, High Pass, Pass, Low Pass, Fail) and percentage of students who failed the clerkship each year (16). The section on remediation/re-demonstration of components in the medicine clerkship consisted of five structured questions. Respondents were asked which clerkship components were required to be passed in order to pass the clerkship. Respondents were also asked what steps were required after students failed a component of the clerkship for the first time and after the second time.

We used descriptive statistics to explain the data. We used the chi-square statistic to examine the association between remediation and faculty rank; responses were dichotomized into remediation versus only re-demonstration (i.e., re-taking the failed component). We also looked at the association between remediation and grading scheme (i.e., Honors, High Pass, Pass, Fail/ABCD/Honors, Pass, Fail) used in the clerkship using the chi-square statistic, as well as between remediation and average percent of students who failed the clerkship in the last academic year using *t*-tests. All analyses were conducted using SAS Enterprise Guide, version 4.3 (SAS Institute, Cary, NC).

## Results

The survey response rate was 73% (85/113). Of these, 51% were male (43/85), 43% female (37/85), and 6% (5/85) did not answer the question. Forty one (48%) were associate professors, 20 (24%) were assistant professors and 18 (21%) were full professors. The majority (88%; 75) were the core medicine clerkship directors.

With regard to medicine clerkship, schools required students to pass the following components: clinical evaluations (97%; 83), NBME subject examination (88%; 76), written assignments (46%; 40), OSCE (41%; 35), in-house written examination (27%; 23), and mini-CEX (22%; 19). Under ‘Other’, respondents listed student presentations, observed history and physical examinations, reflective essays, participation in team-based learning sessions, portfolios, on-line learning modules (e.g., SIMPLE Cases), diagnosis logs, and other quizzes/examinations. Table 1 shows the number of components required to pass the clerkship.

**Table 1:** Number of components required to pass the internal medicine clerkship

Number of components required to pass	Number of clerkships (%) *n*=84^a^
1	1 (1)
2	16 (19)
3	28 (33)
4	20 (24)
5	13 (15)
6	4 (5)
7	1 (1)
8	1 (1)

a One participant had missing responses.

With regard to ‘remedial’ measures required when students failed a component of the clerkship for the first time, 55 schools (64%) simply allowed students to make up (i.e., re-demonstrate) the component, while only 16 (18%) allowed a simple make-up if that component was failed a second time. Twenty-four schools (28%) required additional ward time for a first-time failure of one component, while 49 (57%) required additional ward time for a second-time failure. Fourteen schools (16%) required additional independent study time for a first-time failure of a component, while 9 (10%) required this for a second-time failure. Faculty-supervised study was required by 6 schools (7%) for a first-time failure and by 17 schools (20%) for a second-time failure. Additional assignments were required by 5 schools (6%) for a first-time failure and by 17 schools (20%) for a second-time failure (Table 2).

**Table 2:** After failing a clerkship component for the first and second time, what remediation is required before making up the component failed?

	First time, *N* (%)	Second time, *N* (%)
None; simple make-up component failed	55 (64)	16 (18)
Additional ward time	24 (28)	49 (57)
Additional independent study time	14 (16)	9 (10)
Faculty-supervised study	6 (7)	17 (20)
Additional assignments	5 (6)	9 (10)

When additional remedial time was required, the amounts of time required for remediation for a first-time failure was 1–2 weeks for 4 schools (5%), 3–4 weeks for 20 schools (23%), 5–8 weeks for 5 schools (6%), and greater than 8 weeks for 6 schools (7%). For second-time failures, 3–4 weeks were required by 12 schools (14%), 5–6 weeks by 6 schools (7%), and 7–12 weeks by 26 schools (30%).

There was no association found between presence of remediation and faculty rank of respondents, nor were there associations found between presence of remediation and grading scheme or percent of students who failed the clerkship in the previous academic year.

## Discussion

Most people would agree that a major goal of medical school training is to graduate physicians who are competent. Epstein and Hundert (17) defined professional competence as ‘the habitual and judicious use of communication, knowledge, technical skills, clinical reasoning, emotions, values and reflection in daily practice for the benefit of the individual and community being served’. Assessing competence with this definition in mind requires a multi-faceted approach and is not a simple task.

In a national sample of CDIM, we found that a majority of schools required passing clinical performance evaluations and the NBME subject examination in order to pass the medicine clerkship. There was considerable variability in what other components were required, and 80% of clerkships required three or more components to be passed. In addition, clerkship directors at many schools considered solely re-taking the failed component to be ‘remediation’.

As very few students fail the medicine clerkship (16), relying on clerkship failure as a way to identify students who are not competent is likely insufficient. However, our finding that most clerkships include multiple assessments give us additional opportunities to identify and help struggling learners. We can re-conceptualize failure of a component of the clerkship and not of the entire clerkship as a new way of identifying lack of competence. This identification of deficiencies is the first step of the four-step remediation process described by Hauer et al. (3). Currently, our study suggests when students fail a component of the clerkship for the first time, more than half of schools proceed with a re-take of the component as the next step. Therefore, many clerkships are missing the opportunity to appropriately remediate the learner as they are moving directly to re-demonstration, Step 4 of the Hauer model, without consideration of specifically diagnosing the underlying problem and creating an individualized learning plan, Step 2, or facilitating opportunities for deliberate practice and feedback, Step 3.

We recommend that once a student is identified as not meeting competence through failing a component of the clerkship, it would be beneficial for clerkship directors to reflect and diagnose the underlying problem. For example, a failure of the NBME subject examination could represent lack of medical knowledge, a student who struggles with standardized test taking, an anxiety disorder, or other root causes. The clerkship director may need assistance from others in the medical school with the diagnostic process. After the root cause of the deficiency is identified, an individualized learning plan can be developed. The student then should be provided opportunities for deliberate practice and feedback. In the example of an NBME subject examination failure due to poor medical knowledge, this may involve structured reading with review of performance on sample questions; failure due to poor standardized test-taking may involve a review of test-taking strategies and completing timed tests. Only after successful completion of a remediation plan would students be asked to re-demonstrate via re-taking of the exam.

This same four-step model could be applied to ‘failure’ of other components. If a student has failed clinical performance on the wards, the simple re-demonstration through completing another month on the wards may not be sufficient to truly remediate the student. Once again, this common practice suggests a missed opportunity for remediation. The learner often goes undiagnosed and the fundamental causes of component failure such as deficits in clinical reasoning, struggles with communication skills, or professionalism issues are unaddressed.

In this survey, clerkship directors at many schools considered solely re-taking the failed component to be ‘remediation’. We would argue that while re-demonstration is necessary, it is not a sufficient remediation. We found no correlation between the use of re-demonstration as sole remediation and the academic rank of the clerkship director, the grading scheme of the school or the percentage of failing students at the school. The lack of remediation prior to re-demonstration when a student first fails a component may contribute to the second-time failure of a student on a given component. Our data suggest that it is rare, even with a second component failure, for a remediation strategy to involve a faculty-directed plan. Though it can be argued that successful re-demonstration is the ultimate goal, studies have shown that when students require additional months or semesters to complete required courses, they are significantly more likely to have problems in residency (11). Students who repeatedly fail parts of the clerkship are those who may struggle in subsequent residency training. Perhaps if remediation was required in addition to re-demonstration, we would be more likely to address the fundamental reasons learners continue to struggle.

It is likely that some required components of the medicine clerkship beyond the NBME subject examination assess multiple competency domains. Therefore, a component failure may not clearly map to a particular competency domain. If clerkship directors adopt systems of evaluation that are more clearly matched with competency domains, as are currently being adopted in residency programs, assessment may more clearly assist with identification of specific deficiencies. This could benefit in diagnosis of the learner and development of an appropriate individualized learning plan. Current clerkship assessment systems may mask struggling learners because of compensatory grading schema that allow for strengths in one domain to compensate for deficiencies in other domains.

There are several limitations to this study. Responders were asked what they required when a student did not pass a component and when they responded with only requirements for re-demonstration, we assumed remediation was not required. It is possible that defining re-demonstration and remediation for responders may have led to different responses. We were unable to link responses for remediation strategies with specific component failures if more than one component was required to pass the clerkship. Not all medicine clerkships in the US and Canada have institutional membership in CDIM, although the majority (79%; 113/143) had institutional membership in 2011. This was a cross-sectional survey study subject to response bias.

Medical educators have a societal responsibility to identify trainees who are not competent and only graduate them when they demonstrate competence and readiness to assume the additional responsibilities and functions of the next level of practice. We also have a responsibility to our trainees to best guide them toward achieving needed competence. Through seizing opportunities to identify students who have not reached competence and following the next steps of a remediation program, including diagnosing the deficiency, developing an individualized learning plan, and allowing opportunities for deliberate practice and feedback *prior* to re-demonstration, educators on the medicine clerkship can hopefully give students a better chance at success in their future endeavors.
